# Vascular hemodynamics and blood pressure differences between young and older women

**DOI:** 10.1186/s40885-021-00181-3

**Published:** 2021-11-15

**Authors:** Brantley K. Ballenger, Gary R. Hunter, Gordon Fisher

**Affiliations:** 1 Department of Kinesiology, Mississippi State University, Mississippi State, MS 39762 USA; 2 Department of Nutrition Sciences, University of Alabama at Birmingham, Birmingham, AL 35233 USA; 3 Department of Human Studies, University of Alabama at Birmingham, Birmingham, AL 35233 USA

**Keywords:** Blood pressure, Inflammation, Hemodynamics, Arteries

## Abstract

**Background:**

Cardiovascular disease is one of the main causes of death in the United States, and hypertension is a primary risk factor. Therefore, the primary causes of hypertension need to be identified so they may be addressed for treatment. The purpose of this study was to compare blood pressure with hemodynamic values and identify factors that may explain blood pressure differences between a cohort of healthy normotensive younger and older women.

**Methods:**

Participants were 49 young (age: 33.8 ± 5.9) and 103 old (age: 65.8 ± 4) who were non-hypertensive, had no previous history of heart disease or type 2 diabetes, body mass index less than 30 kg/m^2^, normal electrocardiography response at rest and during exercise, nonsmokers, and no use of medications known to affect cardiovascular or metabolic function. Body composition measured by dual-energy X-ray absorptiometry. Hemodynamic values measured by non-invasive pulse wave velocity through radial artery tonometry. Markers of inflammation measured through blood sample analysis.

**Results:**

Significant differences exist between young and old groups in %fat (*P <* 0.001), systolic blood pressure (SBP) (*P =* 0.001), large artery elasticity (*P =* 0.005), small artery elasticity (*P <* 0.001), systemic vascular resistance (*P =* 0.004), total vascular impedance (*P <* 0.001), estimated cardiac output (*P <* 0.001), and tumor necrosis factor-⍺ (TNF-⍺) (*P <* 0.001). Using ANCOVA the difference in SBP between age groups was no longer significant after adjusting for small artery elasticity (*P <* 0.001) and TNF-⍺ (*P =* 0.041).

**Conclusions:**

These data demonstrate that blood pressure and vascular hemodynamic measures differ significantly between young and old women independent of body composition. Furthermore, these differences may be explained by the inflammation marker TNF-⍺ and/or small artery elasticity.

## Background

Hypertension is a primary risk factor for cardiovascular disease (CVD) and even a slight increase in blood pressure can heighten the risk for developing CVD [1]. Arterial stiffness has been shown to be associated with CVD. Furthermore, it has been shown that functional impairment of the arterial wall can be detected well before symptoms of CVD are present [2, 3]. There are several studies that have also shown a potential link between endothelial dysfunction and arterial stiffness specifically in hypertensive individuals, which suggest that they may be used as a critical tool to evaluate the progression of CVD [4, 5]. The occurrence of arterial stiffness, hypertension, and related cardiovascular diseases is higher in aged populations as compared to younger counterparts [6]. It has been well documented that age may be a risk factor for hypertension and that the prevalence of high blood pressure increases as we age if left untreated [7–9]. Alternatively, previous studies have shown that in menopausal women with endothelial dysfunction, carotid arterial stiffness and epicardial fat thickness are associated with the menopausal transition being independent of age [10]. Thus, it is difficult to determine if high blood pressure and/or arterial stiffness in women is associated with aging, secondary comorbidities, obesity, systemic inflammation, inactivity, or other natural occurrences such as menopause. Therefore, it is important to determine whether central arterial stiffness is a cause or effect of elevated blood pressure [11].

CVD is known as a “silent killer” due to the asymptomatic development starting as early as the first decade of life, thus it is important for medical professionals to identify valid and reliable techniques to assess and track the progression of CVD [12]. Previous studies have shown that hypertension is associated with other cardiovascular risk factors such as arterial elasticity [13]. As mentioned before, similar studies have shown that arterial elasticity will decrease where endothelial dysfunction and hypertension will increase with age [5–7, 9, 11]. An important point to understand about this though, is that this progression may occur differently depending on an individual’s gender, race, or environment. Select studies on hypertension focus on middle-aged and older adult populations [14–16], but other research have demonstrated importance of studying children and young adults due to the increasingly higher prevalence of cardiovascular and metabolic disease risk factors in this population [17–19]. Evidence number of studies have shown associations between tumor necrosis factor-α (TNF-α), and arterial elasticity, arterial inflammation, and endothelial dysfunction [20–23]. Relationships exist between hypertension and age-related arterial stiffness in cohorts of men, and men and women together [1, 5–7, 9, 11], but few studies have been conducted on cohorts of women alone [10, 24, 25]. Women have a significantly higher prevalence of hypertension as compared to men and have less success in lowering blood pressure when treated pharmacologically which can confound studies on older adults [1, 9, 26].

Therefore, it is important to compare blood pressure with vascular hemodynamic values between younger and older women that are healthy and normotensive. Thus, the primary purpose of this study was to identify factors that may explain blood pressure differences between a cohort of healthy normotensive younger and older women. The result of which would identify if these blood pressure differences may be explained by differences in markers of inflammation, body composition, or arterial elasticity.

## Methods

### Participants

This is a secondary analysis of two studies designed to evaluate free living energy expenditure. This data analysis consisted of 49 females aged 21–46, and 103 females aged 60+. The two groups of females were divided into young and old groups based on age. Inclusion requirements for participants were non-hypertensive (systolic blood pressure [SBP] < 140 or diastolic blood pressure [DBP] < 90 mmHg), no previous history of heart disease or type 2 diabetes, normal electrocardiogram (EKG) response at rest and during exercise, nonsmokers, and no use of medications known to affect cardiovascular or metabolic function. Preliminary screening for study inclusion also included a physical examination, dual-energy X-ray absorptiometry, and a 12-lead EKG. Participants were excluded from the study if they were hypertensive or dual-energy X-ray absorptiometry assessment revealed osteoporosis. The study was approved by the Institutional Review Board for Human Use at the University of Alabama at Birmingham. All women provided informed consent prior to participating in the study.

### Body composition

Body composition was measured by dual-energy X-ray absorptiometry (Prodigy; Lunar Radiation, Madison, WI). The scans were analyzed with the use of ADULT software, version 1.33 (Lunar Radiation).

### Arterial elasticity evaluation

Hemodynamic values such as SBP and DBP, large artery elasticity (LAE), small artery elasticity (SAE), estimated cardiac output (ECO), systemic vascular resistance (SVR), and total vascular impedance (TVI) were all measured by non-invasive pulse wave velocity analysis through radial artery tonometry (HDI/Pulse Wave TM CR-2000, Hypertension Diagnostics, Eagan, MN). Participants were placed in the seated position, with a solid-state pressure transducer array (tonometer) placed over the radial artery of the dominant arm to record the pulse contour. The waveform from the tonometer was calibrated by the oscillometric method. Once a stable measurement was achieved, a 30 s analog tracing of the radial waveform was digitized at 200 samples per second. Before, during, and after the waveform assessment, an automated oscillatory blood pressure measurement was taken on the contralateral arm. The first maximum waveform observed represented the action of the arteries following cardiac ejection and reflects the large arteries, whereas the second rebound wave reflects compliance of the smaller arteries. SVR was calculated as the mean arterial pressure divided by the ECO. TVI was determined from the modified Windkessel model evaluated at the frequency of the measured heart rate at rest [20].

### Markers of inflammation

Systemic inflammation was measured by serum levels of TNF-α, interleukin-6 (IL-6), and C-reactive protein (CRP) through blood sample analysis. TNF-α was analyzed using the high-sensitivity ELISA kit (Quantikine HSTA00C, R&D Systems, Minneapolis, MN). IL-6 was assayed using the high-sensitivity ELISA kit (Quantikine HS600B, R&D Systems). CRP was assayed with the high-sensitivity ELISA kit (030–9710 s, ALPCO, Windham, NH).

### Statistical analysis

Data was analyzed using the Statistical Package for the Social Sciences (SPSS, version 26.0, Chicago, IL). Means and standard deviations for anthropomorphic and hemodynamic measures, LAE, SAE, SVR, TVI, ECO, and proinflammatory cytokine levels, CRP, TNF-⍺, and IL-6 were reported and assessed by one-way analysis of variance (ANOVA) with a significance value of *P* <  0.05. Simple Pearson correlations were also used to examine associations of weight, BMI, %fat, SBP and DBP, as well as LAE and SAE, SVR and TVI, ECO, and the proinflammatory cytokine levels CRP, TNF-⍺, and IL-6 for the young and old groups. An analysis of covariance (ANCOVA) was performed to explain differences between SBP and age with %fat, LAE, SAE, and levels of TNF-⍺, CRP, and IL-6.

## Results

Differences between body composition, vascular measures, and markers of inflammation are presented in Table 1. Significant differences between young and old were found in %fat, SBP, LAE, SAE, SVR, TVI, ECO, and TNF-⍺ (*P <* 0.05). No significant differences were observed between the age groups in weight, height, BMI, DBP, CRP or IL-6.

**Table 1:** Mean ± standard deviation for total participants and comparing young and old

	Total	Young	Old	*P-*value
	(*n* = 152)	(*n* = 49)	(*n* = 103)	
Age (yr)*	54.51 ± 16.09	33.83 ± 5.88	65.81 ± 3.96	**< 0.001**
Weight (kg)	74.72 ± 12.60	76.55 ± 14.70	73.85 ± 11.44	0.218
Height (cm)	165.20 ± 5.82	165.55 ± 5.96	165.04 ± 5.78	0.617
BMI (kg/m^2^)	27.40 ± 4.58	27.91 ± 4.98	27.16 ± 4.38	0.353
DXA Fat (%)*	41.47 ± 6.44	38.66 ± 6.60	42.76 ± 5.96	**< 0.001**
SBP (mmHg)*	123.55 ± 13.92	118.11 ± 11.97	125.97 ± 14.10	**0.001**
DBP (mmHg)	69.84 ± 9.28	70.90 ± 8.20	69.37 ± 9.73	0.362
LAE*	13.97 ± 5.04	15.67 ± 5.23	13.13 ± 4.75	**0.005**
SAE*	4.96 ± 2.52	6.99 ± 2.50	3.96 ± 1.85	**< 0.001**
SVR*	1519.89 ± 352.21	1398.23 ± 457.43	1580.05 ± 269.66	**0.004**
TVI*	151.79 ± 47.80	121.69 ± 38.62	166.68 ± 44.95	**< 0.001**
ECO*	4.979 ± 0.74	5.30 ± 0.87	4.79 ± 0.60	**< 0.001**
CRP	2.71 ± 2.97	2.50 ± 2.86	2.91 ± 3.09	0.515
TNF-⍺*	5.19 ± 2.37	3.45 ± 0.89	6.61 ± 2.24	**< 0.001**
IL-6	1.84 ± 2.34	1.74 ± 3.06	1.92 ± 1.56	0.72

Measures include age, weight, height, *BMI* body mass index, *DXA Fat* body fat, *SBP* systolic blood pressure, *DBP* diastolic blood pressure, *LAE* large artery elasticity, *SAE* small artery elasticity, *SVR* systemic vascular resistance, *TVI* total vascular impedence, *ECO* estimated cardiac output, *CRP* C-reactive protein, *TNF-α* tumor necrosis factor-α and *IL-6* interleukin-6.

*P <  0.05 in ANOVA between young and old groups

Results from ANCOVA are shown in Table 2. Model 1 shows the probability for SBP differences in SBP after adjusting for age. Models 2–7 demonstrate the ANCOVA for SBP after adjusting for age, and a third variable. These data demonstrate that SBP differences observed between young and old were independent of %fat, LAE, CRP, and IL-6. However, the age differences in SBP may be partly explained by differences in serum TNF-⍺ and/or SAE.

**Table 2:** Systolic blood pressure (SBP) models after adjusting for potential cofounders

Model		Independent variable	*P-*value
1	**SBP**	Age	**< 0.001**
2	SBP	Age	**0.014**
		% Fat DXA	0.072
3	SBP	Age	**0.017**
		LAE	**< 0.001**
4	SBP	Age	0.465
		SAE*	**< 0.001**
5	SBP	Age	0.235
		TNF-α*	**0.041**
6	SBP	Age	**0.001**
		CRP	0.366
7	SBP	Age	**0.001**
		IL-6	0.361

Independent cofounding variables include age, *% Fat DXA* body fat, *LAE* large artery elasticity, *SAE* small artery elasticity, *CRP* C-reactive protein, *TNF-α* tumor necrosis factor-α and *IL-6* interleukin-6.

*P < 0.05 in ANCOVA after adjusting for age

Pearson correlation analysis between variables are shown in Table 3. For both the young and old groups combined, there was a positive association with BMI and SBP, DBP, ECO, and CRP. BMI was negatively associated with SVR (*P <* 0.05). SBP was positively associated with DBP, SVR, and TVI, while an inverse association was found between SBP and LAE and SAE (*P <* 0.05). LAE and SAE were positively associated with ECO, and inversely associated with SVR and TVI (*P <* 0.05). TNF-⍺ was positively associated with %fat, SBP, SVR and TVI, but an inverse association was seen between TNF-⍺ and LAE, SAE, and ECO (*P <* 0.05).

**Table 3:** Pearson correlation table total (young and old)

	Weight	BMI	DXA Fat	SBP	DBP	LAE	SAE	SVR	TVI	ECO	CRP	TNF-⍺	IL-6
Weight	1	0.902	0.645	0.24	0.232	−0.07	0.196	−0.292	− 0.003	0.639	0.208	0.021	0.025
		**< 0.0001**	**< 0.0001**	**0.003**	**0.005**	0.417	**0.022**	**0.001**	0.974	**< 0.0001**	**0.049**	0.847	0.817
BMI		1	0.728	0.232	0.276	−0.115	0.068	−0.239	0.044	0.533	0.227	0.045	0.144
			**< 0.0001**	**0.005**	**0.001**	0.181	0.433	**0.005**	0.607	**< 0.0001**	**0.031**	0.671	0.28
DXA Fat			1	0.224	0.115	−0.22	−0.189	−0.109	0.216	0.295	0.29	0.356	0.116
				**0.007**	0.169	**0.01**	**0.027**	0.207	**0.011**	**0.001**	**0.006**	**< 0.0001**	0.271
SBP				1	0.629	−0.373	− 0.439	0.371	0.441	−0.049	0.077	0.386	−0.065
					**< 0.0001**	**< 0.0001**	**< 0.0001**	**< 0.0001**	**< 0.0001**	0.57	0.476	**< 0.0001**	0.54
DBP					1	−0.119	−0.164	0.363	0.063	−0.024	− 0.06	0.168	− 0.117
						0.167	0.056	**< 0.0001**	0.464	0.781	0.579	0.114	0.274
LAE						1	0.36	−0.416	−0.796	0.175	−0.17	− 0.413	0.01
							**< 0.0001**	**< 0.0001**	**< 0.0001**	**0.041**	0.12	**< 0.0001**	0.929
SAE							1	−0.522	−0.535	0.444	−0.088	− 0.466	− 0.097
								**< 0.0001**	**< 0.0001**	**< 0.0001**	0.423	**< 0.0001**	0.37
SVR								1	0.587	−0.748	−0.039	0.347	−0.05
									**< 0.0001**	**< 0.0001**	0.726	**0.001**	0.644
TVI									1	−0.353	0.213	0.575	0.025
										**< 0.0001**	**0.05**	**< 0.0001**	0.815
ECO										1	0.092	− 0.336	− 0.073
											0.403	**0.001**	0.504
CRP											1	0.197	0.024
												0.062	0.824
TNF-⍺												1	0.061
													0.559
IL-6													1

Measures used for correlations with total participants include weight, *BMI* body mass index, *DXA Fat* body fat, *SBP* systolic blood pressure, *DBP* diastolic blood pressure, *LAE* large artery elasticity, *SAE* small artery elasticity, *SVR* systemic vascular resistance, *TVI* total vascular impedence, *ECO* estimated cardiac output, *CRP* C-reactive protein, *TNF-α* tumor necrosis factor-α and *IL-6* interleukin-6

Tables 4 and 5 represent the correlation table for young participants and older participants respectively. In the older group BMI was positively associated with DBP and SAE while an inverse association was seen between BMI and LAE and SVR (*P* < 0.05). BMI was not seen to have these same associations in the young group. SBP was negatively associated with LAE and SAE (*P <* 0.05) in the old group but this relationship was only seen between SBP and LAE in the young group. %Fat was positively associated with CRP in the young group, while %fat was positively associated with IL-6 in the older group (*P* < 0.05). TNF-⍺ was significant with SBP, DBP, LAE, SVR, and TVI in the older group. This significance was not seen in the young group suggesting that inflammation may have a greater affect in the older population.

**Table 4:** Pearson correlation table young

	Weight	BMI	DXA Fat	SBP	DBP	LAE	SAE	SVR	TVI	Est. CO	CRP	TNF-⍺	IL-6
Weight	1	0.923	0.701	0.388	0.196	0.053	−0.066	−0.293	− 0.13	0.688	0.22	0.015	−0.101
		**< 0.0001**	**< 0.0001**	**0.008**	0.198	0.73	0.666	**0.05**	0.394	**< 0.0001**	0.157	0.924	0.53
BMI		1	0.76	0.405	0.202	−0.012	−0.278	−0.233	− 0.022	0.586	0.303	0.036	0.028
			**< 0.0001**	**0.006**	0.183	0.935	0.065	0.124	0.886	**< 0.0001**	**0.048**	0.822	0.863
DXA Fat			1	0.146	−0.077	−0.047	−0.199	− 0.261	0.02	0.48	0.405	0.246	−0.06
				0.34	0.615	0.762	0.189	0.084	0.897	**0.001**	**0.008**	0.121	0.708
SBP				1	0.834	−0.447	−0.281	0.193	0.341	0.245	0.056	−0.201	−0.2
					**< 0.0001**	**0.002**	0.062	0.204	**0.036**	0.105	0.726	0.214	0.217
DBP					1	−0.328	−0.25	0.376	0.223	0.052	−0.135	−0.141	− 0.2
						**0.028**	0.097	**< 0.011**	0.141	0.734	0.402	0.387	0.216
LAE						1	0.351	−0.478	−0.772	0.242	− 0.06	− 0.076	0.042
							**0.035**	**0.001**	**< 0.0001**	0.11	0.711	0.639	0.795
SAE							1	−0.368	−0.484	−0.191	− 0.118	0.113	− 0.109
								**0.013**	**0.001**	0.209	0.462	0.488	0.503
SVR								1	0.771	−0.769	−0.08	0.161	−0.01
									**< 0.0001**	**< 0.0001**	0.619	0.321	0.951
TVI									1	−0.554	0.188	0.093	0.045
										**< 0.0001**	0.24	0.568	0.784
Est. CO										1	0.134	−0.178	−0.122
											0.403	0.271	0.454
CRP											1	0.121	−0.024
												0.451	0.879
TNF-⍺												1	−0.108
													0.5
IL-6													1

Measures used for correlations with young participants include weight, *BMI* body mass index, *DXA Fat* body fat, *SBP* systolic blood pressure, *DBP* diastolic blood pressure, *LAE* large artery elasticity, *SAE* small artery elasticity, *SVR* systemic vascular resistance, *TVI* total vascular impedence, *ECO* estimated cardiac output, *CRP* C-reactive protein, *TNF-α* tumor necrosis factor-α and *IL-6* interleukin-6

**Table 5:** Pearson correlation table old

	Weight	BMI	DXA Fat	SBP	DBP	LAE	SAE	SVR	TVI	Est. CO	CRP	TNF-⍺	IL-6
Weight	1	0.888	0.715	0.204	0.252	−0.183	0.457	0.292	0.103	0.632	0.22	0.144	0.289
		**< 0.0001**	**< 0.0001**	**0.04**	**0.011**	0.083	**< 0.0001**	**0.005**	0.332	**< 0.0001**	0.138	0.318	**0.042**
BMI		1	0.798	0.18	0.309	−0.211	0.339	−0.243	0.122	0.519	0.174	0.14	0.37
			**< 0.0001**	0.071	**0.002**	**0.045**	**0.001**	**0.02**	0.248	**< 0.0001**	0.243	0.331	**0.008**
DXA Fat			1	0.153	0.243	−0.217	0.172	−0.173	0.102	0.452	0.175	0.187	0.385
				0.126	**0.014**	**0.038**	0.104	0.101	0.335	**< 0.0001**	0.24	0.195	**0.006**
SBP				1	0.596	−0.274	−0.331	0.415	0.378	−0.033	0.129	0.351	−0.2
					**< 0.0001**	**0.009**	**0.001**	**< 0.0001**	**< 0.0001**	0.755	0.394	**0.013**	0.217
DBP					1	−0.328	−0.25	0.376	0.223	0.052	−0.135	0.433	−0.2
						**0.028**	0.097	**< 0.011**	0.141	0.734	0.402	**0.002**	0.216
LAE						1	0.351	−0.478	−0.772	0.242	−0.06	−0.426	0.042
							**0.035**	**0.001**	**< 0.0001**	0.11	0.711	**0.003**	0.795
SAE							1	−0.368	−0.484	−0.191	− 0.118	− 0.179	−0.109
								**0.013**	**0.001**	0.209	0.462	0.235	0.503
SVR								1	0.771	−0.769	−0.08	0.386	−0.01
									**< 0.0001**	**< 0.0001**	0.619	**0.008**	0.951
TVI									1	−0.554	0.188	0.416	0.045
										**< 0.0001**	0.24	**0.004**	0.784
Est. CO										1	0.134	−0.144	−0.122
											0.403	0.34	0.454
CRP											1	0.279	−0.024
												0.057	0.879
TNF-⍺												1	−0.108
													0.5
IL-6													1

Measures used for correlations with old participants include weight, *BMI* body mass index, *DXA Fat* body fat, *SBP* systolic blood pressure, *DBP* diastolic blood pressure, *LAE* large artery elasticity, *SAE* small artery elasticity, *SVR* systemic vascular resistance, *TVI* total vascular impedence, *ECO* estimated cardiac output, *CRP* C-reactive protein, *TNF-α* tumor necrosis factor-α and *IL-6* interleukin-6

## Discussion

Due to the rise in prevalence of hypertension in the older population [6], and the relationship that is present between hypertension and arterial stiffness [12], the purpose of this study was to compare blood pressure with hemodynamic values of young and old normotensive participants to try and identify potential factors that predict the onset of developing hypertension and other CVDs. Factors that may prevent or treat hypertension are becoming increasingly important due to the prevalence of hypertension rising rapidly throughout the aging population [1] and limited societal awareness and response [7]. The primary findings of the study were that blood pressure and vascular hemodynamic measures differ between young and old women independent of body composition. Additionally, blood pressure differences between age groups were not explained by differences in LAE, CRP, or IL-6. However, these differences were partially explained by differences in SAE and TNF-⍺. Overall, these findings suggest that systemic inflammation as well as small arterial elasticity differences between younger and older healthy normotensive women may explain some age-related differences in blood pressure.

Studies have shown body composition measures to be strong predictors of arterial stiffness in young adults [17] and a regression in arterial stiffness has been seen with weight loss in both obese and nonobese individuals [18]. Participants for this study were matched for height and weight, so that BMI would not confound blood pressure measures. Participants were also excluded if they had osteoporosis. Thus, no significant differences were seen in height, weight or BMI, between the young and older participants. There was, however, a significant difference in %fat observed between the two groups. It is known that aging is associated with a shift of fat from the periphery to a more visceral fat distribution, though less is known about why this shift in fat distribution occurs [27]. This could explain the higher prevalence of body fat in the older group. This study saw that age related differences in blood pressure were independent of %fat, but since body fat percentage is associated with systemic inflammation [27], this could be one reason why we see increased TNF-⍺ levels. Higher %fat could equate to higher inflammation thus resulting in the higher SBP in the older group. More research is needed to assess if this increase in body fat is responsible for increased inflammation.

The ability of the arteries to perform properly and maintain regular blood flow and pressure can be hindered due to aging or other factors such as kidney disease, diabetes, atherosclerosis [28, 29] or hormonal changes during pregnancy or menopause [30]. Significant differences in arterial elasticity were seen between the young and old participants, and analysis of covariance showed that age differences in blood pressure could possibly be explained by SAE but not by LAE. This difference in arterial elasticity between the groups could partially be explained by increases in inflammation, which is known to decrease elasticity and affect endothelial health [16, 19]. This increased inflammation could be what causes SAE to explain age related differences in blood pressure in this study while LAE does not. Previous research has also shown that females develop higher distensability in their arteries while males develop stiffer arteries post puberty [31], this could explain some gender differences that are present with blood pressure [32]. Further research should look at the relationship to determine why smaller arteries may be affected by inflammation more than larger arteries and also further explore the gender differences present in blood pressure and arterial elasticity and distensability.

TNF-⍺, IL-6, and CRP have all been shown to be associated with CVD, and of those, TNF-⍺ has been shown to be the most correlated with arterial elasticity, vascular resistance, and blood pressure [20]. Of the three proinflammatory cytokines observed in this study, TNF-⍺ may explain age differences in blood pressure while CRP and IL-6 remained independent of age differences. This increase in inflammation, brought on by age or other factors such as an increase in body fat, could also result in the stiffening of arteries leading to cardiovascular or metabolic disease [15, 33]. Correlations with levels of TNF-⍺ and blood pressure were only seen in the older group. This could be due to the younger group being overall healthier and simply not having significant inflammation present.

The association between aging and blood pressure is complex. It is well known that aging [7–9] and other factors such as excess food intake [18, 33], shift in fat distribution [27], and lack of exercise [14], affect vascular hemodynamics but what are less known are factors that decrease this progression. This emphasizes the importance of studies with the goal of continuing to learn about this association and ways to decrease the prevalence of hypertension [29]. Previous studies have shown that exercise could be a factor that decreases the progression of hypertension. Aerobic exercise has been shown to elicit an increase in central aortic distensibility while resistance exercise may produce vasodialators not present after aerobic exercise [34, 35], but less it known about why this takes place. The present study suggests that increased TNF-⍺ and decreased SAE in older women could be a main contributing factor in the increase in SBP. An age-related increase in visceral fat over time is also known to contribute to an increase in inflammation [27], which then can lead to reduced small artery health. This decreased arterial health can then lead to an increase in blood pressure and may be the cause of cardiovascular or metabolic diseases in older women who are not overweight or obese. Because of this, it is possible that systemic inflammation and arterial elasticity differences between younger and older women, who are relatively matched in body composition, might explain the differences in blood pressure measures.

Overall strengths of this study include 1) strict inclusion requirements, 2) a large population size of young (*n* = 49) and old (*n* = 103) women, and 3) the use of state-of-the-art methods to assess body composition, hemodynamic measures, and inflammation markers. This study also provides clinical significance for the development of gender specific hypertension intervention. As previously stated, to the authors knowledge this is one of the first studies to look at differences in blood pressure and vascular measures in women alone. This study offers insight for health professionals to better monitor hypertension emergence in women by detecting the above mentioned risk factors early. The primary limitation of this study was the lack of aerobic fitness level inclusion, which could play a role in the prevalence of inflammation and arterial elasticity. Future research would be needed to investigate the relationship between fitness levels, systemic inflammation, and blood pressure differences between younger and older women.

## Conclusions

These data demonstrate that blood pressure and vascular hemodynamic measures differ between young and old women independent of body composition. These differences may be at least partially explained by the inflammatory marker TNF-⍺ and SAE. Therefore, monitoring of these values in women as they age may help to detect the risk for emergence of hypertension.

## Data Availability

The datasets used and/or analysed during the current study are available from the corresponding author on reasonable request.

## Abbreviations

CRP: C-reactive protein
CVD: Cardiovascular disease
DBP: Diastolic blood pressure
ECO: Estimated cardiac output
EKG: Electrocardiogram
IL-6: Interleukin-6
LAE: Large artery elasticity
SAE: Small artery elasticity
SBP: Systolic blood pressure
SVR: Systemic vascular resistance
TNF-α: Tumor necrosis factor-α
TVI: Total vascular impedance
